# Sub-acute Changes on MRI Measures of Cerebral Blood Flow and Venous Oxygen Saturation in Concussed Australian Rules Footballers

**DOI:** 10.1186/s40798-022-00435-w

**Published:** 2022-04-01

**Authors:** David K. Wright, Terence J. O’Brien, Sandy R. Shultz

**Affiliations:** 1 Department of Neuroscience, The Alfred Centre, Central Clinical School, Monash University, 99 Commercial Road, Melbourne, VIC 3004 Australia; 2 Department of Medicine, The Royal Melbourne Hospital, The University of Melbourne, Parkville, VIC 3052 Australia

**Keywords:** Mild traumatic brain injury, Hypometabolism, Return-to-play, Quantitative susceptibility mapping, Neuroimaging

## Abstract

**Background:**

Sports-related concussion (SRC) is common in collision sport athletes. There is growing evidence that repetitive SRC can have serious neurological consequences, particularly when the repetitive injuries occur when the brain has yet to fully recover from the initial injury. Hence, there is a need to identify biomarkers that are capable of determining SRC recovery so that they can guide clinical decisions pertaining to return-to-play. Cerebral venous oxygen saturation (SvO_2_) and cerebral blood flow (CBF) can be measured using magnetic resonance imaging (MRI) and may provide insights into changing energy demands and recovery following SRC.

**Results:**

In this study we therefore investigated SvO_2_ and CBF in a cohort of concussed amateur Australian Football athletes (i.e., Australia’s most participated collision sport). Male and female Australian footballers (*n* = 13) underwent MRI after being cleared to return to play following a mandatory 13-day recovery period and were compared to a group of control Australian footballers (*n* = 16) with no recent history of SRC (i.e., > 3 months since last SRC). Despite the concussed Australian footballers being cleared to return to play at the time of MRI, we found evidence of significantly increased susceptibility in the global white matter (*p* = 0.020) and a trend (*F*_5,21_ = 2.404, *p* = 0.071) for reduced relative CBF (relCBF) compared to the control group. Further, there was evidence of an interaction between sex and injury in straight sinus susceptibility values (*F*_1,25_ = 3.858, *p* = 0.061) which were decreased in female SRC athletes (*p* = 0.053). Of note, there were significant negative correlations between straight sinus susceptibility and relCBF suggesting impaired metabolic function after SRC.

**Conclusions:**

These findings support the use of quantitative susceptibility mapping (QSM) and relCBF as sensitive indicators of SRC, and raise further concerns related to SRC guidelines that allow for return-to-play in less than two weeks.

## Key Points

- Cerebral venous oxygen saturation (SvO_2_) and cerebral blood flow (CBF) can be measured using MRI and may provide insights into changing energy demands and recovery following sports related concussion (SRC).
- Although clear to return to play at the time of MRI, we found significant changes in CBF and susceptibility suggesting increased cerebral vulnerability in athletes following SRC.
- These findings raise further concerns related to SRC guidelines that allow for return-to-play in less than 2 weeks.

## Background

Sports-related concussion (SRC), like other mild traumatic brain injuries (mTBI), is caused by the rapid acceleration or deceleration of the brain which stretches the cytoskeleton and initiates a cascade of ionic fluxes and the indiscriminate release of the excitatory neurotransmitter glutamate. An energy crisis ensues as ATP-powered membrane pumps attempt to restore the ionic balance [1]. Acute hyperglycolysis is followed by a hypometabolic period that can last for days after injury—potentially increasing the risk of exacerbated neurological outcomes following a subsequent injury [2]. As such, athletes are now held out of play until they are at least asymptomatic to mitigate risk, although there is still no consensus on how long is required for, or whether symptom status accurately reflects, neurobiological recovery [3].

A number of promising biomarkers have been developed with the aim of objectively determining recovery. Magnetic resonance imaging (MRI) techniques including magnetic resonance spectroscopy [4] and diffusion weighted imaging [5–8] have been used to investigate a range of often subtle physiological cascades that follow SRC. Quantitative susceptibility mapping (QSM) is a comparatively new technique that utilizes MR phase images to map tissue susceptibility (*χ*) [9]. QSM is sensitive to diamagnetic and paramagenetic biomaterials including, among others, ferritin, hemosiderin, myelin and calcium [9, 10] and has shown promise in assessing grey and white matter susceptiblity changes acutely following SRC [11, 12].

Recently, QSM has also been used to investigate cerebral venous oxygen saturation (SvO_2_) and in doing so, potentially provide insights into changing energy demands following injury [13]. SvO_2_ is measured using quantitative susceptibility mapping (QSM) which utilizes MR phase images to map tissue susceptibility (*χ*) [9]. During brain metabolism, the release of O_2_ from diamagnetic (*χ* < 0) hemoglobin produces paramagenetic (*χ* > 0) deoxyhemoglobin. In mTBI, decreases in venous susceptibility and hence increased SvO_2_ have been observed acutely following injury, potentially reflecting post-injury hypometabolism [13, 14].

Increased SvO_2_ may also reflect increased cerebral blood flow (CBF) that exceeds cerebellar energy demands [14]. Like SvO_2_, CBF can also be estimated non-invasively using MRI [15] and perturbations in CBF have been observed after mTBI, including SRC [16–19]. However, whether there is a relationship between decreased venous susceptibility (i.e., increased SvO_2_) and increased CBF has yet to be determined. Furthermore, previous SvO_2_ and CBF studies in SRC have included limited sub-acute recovery times and have focused on North American sports (e.g., American football). No studies to date have examined SvO_2_ and CBF in Australian rules footballers (i.e., Australia’s most participated collision sport), and an understanding of sub-acute recovery at approximately two weeks post-SRC is of particular relevance to return to play decisions (i.e., has the brain recovered) considering that professional and amateur Australian rules football leagues mandate a minimum 12-day recovery time. Therefore, here we acquired QSM and CBF images in a cohort of amateur Australian rules football athletes to test the hypotheses that tissue susceptibility changes and increased SvO_2_ would be present at 13 days post-SRC, and that SvO_2_ and CBF would be correlated.

## Methods

### Ethics Approval and Consent to Participate

Study procedures were approved by the Melbourne Health Human Ethics Institutional Review Board (#2015.012), were in accordance with The Code of Ethics of the World Medical Association (Declaration of Helsinki) for experiments involving humans, and all participants provided written informed consent prior to the study.

### Participants

Amateur Australian Football athletes were recruited from clubs in Melbourne, Victoria, between 2018 and 2020. Control athletes (9 males, 7 females), with no recent SRC in the past 3 months, were compared to athletes with SRC (7 males, 6 females). Athletes were withheld from contact sports for 2 weeks following injury, and MRI scanning was performed on day 13 (the day before they returned to play). Athletes were interviewed to determine their history of concussion (HoC), neurosurgery, or major psychiatric disturbances.

### SRC Diagnosis

SRC was diagnosed by the team physician based on sideline and/or postgame assessment that included the Sports Concussion Assessment Tool (SCAT). When available, video evidence was also reviewed for immediate signs of SRC.

### MRI Acquisition and Processing

Neuroimaging was performed with a Siemens 3 T Prisma MRI. 3D T_1_-weighted images were acquired using an MPRAGE sequence with repetition time (TR) = 2400 ms, echo time (TE) = 2.24 ms, inversion time (TI) = 1060 ms, flip angle = 8°, and resolution = 0.8 × 0.8 × 0.8 mm^3^. T_2_^*^-weighted multi-echo images were acquired with TR = 24 ms, TE = 4.26 ms, ΔTE = 2.31 ms; flip angle = 20°; resolution = 0.83 × 0.83 × 0.83 mm^3^ and flow compensation turned on. Relative CBF (relCBF) was measured using a 2D PICORE pulsed arterial spin labeling (PASL) product sequence with TR = 2500 ms, TE = 11 ms, PICORE Q2T perfusion mode with 101 dynamics (50 pairs of tag and control measurements and one M_0_ image), bolus duration = 1600 ms, inversion time = 1800 ms, in-plane resolution = 3 × 3 mm^2^, 14 slices of thickness = 6 mm with slice spacing 7.5 mm and motion correction turned on. This commercially available sequence has good within and between session reproducibility [20] and has been selected as the Siemens MRI sequence for a large-scale multisite study of concussion [21].

Advanced Normalisation Tools (ANTs) was used to create a study-specific template image from each subject’s T_1_-weighted image [22]. The template image was registered to MNI space to facilitate segmentation with regions of interest (ROIs) defined by the Harvard–Oxford Subcortical Structural Atlas (included as part of the FMRIB Software Library). Intra-subject registration of motion corrected ASL and T_2_^*^-weighted images to the T_1_-weighted image was performed using an affine registration, also with ANTs and the Harvard–Oxford Subcortical Structural Atlas transformed into subject space. Atlas images and Siemen’s reconstructed relCBF images were read into MATLAB (R2021a, MathWorks, Natick, Massachusetts) and the mean value determined for 5 ROIs: the right and left cerebral cortex, the right and left white matter, and the basal ganglia (comprising bilateral caudate, putamen, accumbens and pallidum regions).

QSM images were reconstructed from Siemen’s magnitude and phase images using nonlinear dipole inversion [23] and MATLAB. To investigate for brain-wide changes in gray matter and white matter susceptibility we generated global gray and white matter masks based on an automated method that incorporates a stability mask [11, 12]. Global grey and white matter masks were generated by first excluding voxels with R_2_^*^ less than 1 ms^−1^ and greater than 65 ms^−1^ to remove large vessels such as the straight sinus from the final gray matter mask. A stability mask was then constructed by computing coefficients of variability with an upper threshold of 0.8. White and gray matter were subsequently defined as *χ* < − 0.03 ppm and *χ* > 0.05 ppm, respectively and mean susceptibility values were then calculated for each [11, 12].

QSM images were also used to assess SvO_2_. Maximum intensity projection (MIP) images were reconstructed over 10 sagittal slices and the mean susceptibility measured in the straight sinus, a vessel known to be affected after mTBI [13]. Finally, correlations were performed between straight sinus susceptibility and relCBF values to assess for a relationship between regional CBF and increased SvO_2_ (i.e., decreased susceptibility), as well as between symptom number and symptom severity and regional measures of relCBF, global grey and white matter QSM values and SvO_2_.

### Statistical Testing

Participant demographics were assessed using a 2-way ANOVA for age, education, participation in collision sports, and previous concussions with injury and sex as between subject factors. Statistical testing of symptom number and symptom severity, relCBF, and whole-brain gray and white matter susceptibility measures, were performed with a 2-way multivariate ANOVA (MANOVA) with injury and sex as between subject factors. Statistical testing of a history of multiple concussions was performed with a χ^2^ test. Susceptibility measures in the straight sinus were analyzed using a two-way ANOVA, with injury and sex as between subject factors. Bonferroni corrected post-hoc comparisons were performed when appropriate. To investigate the relationship between symptom number and severity scores and the measured relCBF and susceptibility values, and between straight sinus susceptibility and relCBF values, Pearson correlation analyses were performed. All statistical testing was performed using SPSS software (version 27.0, IBM Corp, Armonk, NY) with significance set at *p* < 0.05. Multiple comparison correction of correlation analyses were performed using the Benjamini–Hochberg false discovery rate correction set to 10%.

## Results

### Participant Demographics Revealed Significant Sex and Injury Effects

Analysis of participant demographics revealed that males had played more years of collision sport (*p* = 0.002) and had a greater number of previous concussions (*p* = 0.027), than their female counterparts. There were significant interactions between sex and SRC for both symptom number and symptom severity. While male SRC athletes tended to have more symptoms and greater symptom severity, the opposite was true in females. In this cohort, female control athletes had both a greater number of symptoms (*p* = 0.016) and symptom severity (*p* = 0.031) than their SRC counterparts.

### A Trend Towards Persisting Hypoperfusion After SRC

**Fig. 1:**
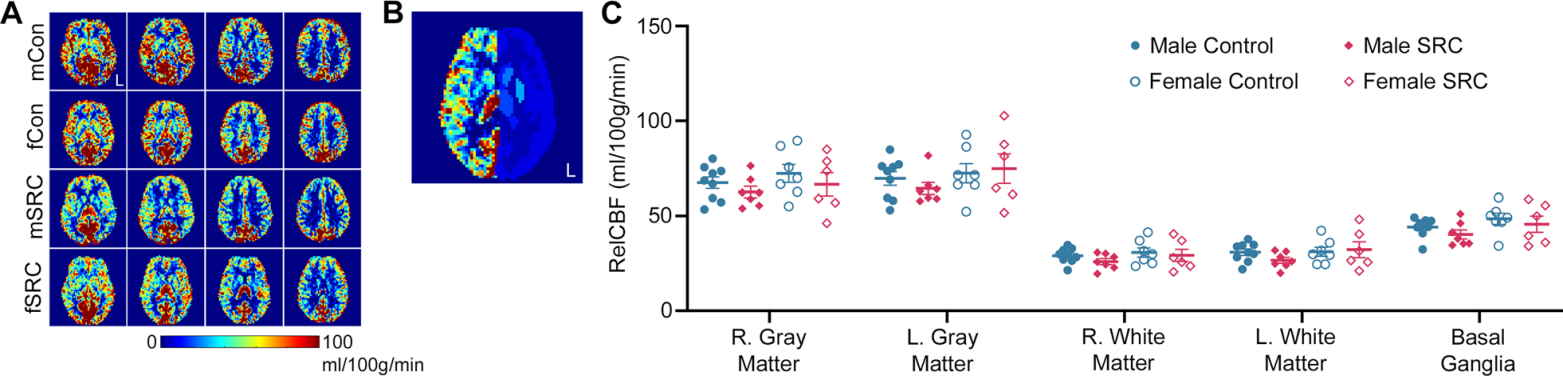
Decreased relative cerebral blood flow (relCBF) 14 days after SRC. **A** Representative relCBF images from one male control (mCon), female control (fCon), male SRC (mSRC), and female SRC (fSRC) athlete. Four slices are shown for each representative athlete. Colorbar shows relCBF values and ‘L’ indicates left side of brain. **B** Representative relCBF image overlaid with co-registered Harvard–Oxford Subcortical Structural Atlas. **C** Median relCBF values plotted for 5 ROIs. Two-way MANOVA revealed a significant main effect of injury with concussed athletes having significantly decreased relCBF (*p* = 0.039). relCBF = relative cerebral blood flow, SRC = sports related concussion

Representative relCBF images are shown in Fig. 1A. We measured mean relCBF in 5 ROIs including the right and left cerebral cortex, the right and left white matter, and the basal ganglia (Table 1). Registered Harvard–Oxford Subcortical Structural Atlas ROIs (left side only) are shown overlaid on the left hemisphere of one representative subject in Fig. 1B. Two-way MANOVA demonstrated a trend for decreased relCBF in athletes with SRC (*F*_5,21_ = 2.404, *p* = 0.071, Wilks’ Λ = 0.636, Fig. 1C). There was no interaction between sex and injury and no main effect of sex (both, *p* > 0.05) (Table 2).

**Table 1:** Participant demographics

	Male		Female		*p* value		
	Control	SRC	Control	SRC	Sex	SRC	Sex × SRC
*n*	9	7	7	6			
Age	24.67 ± 0.83	22.14 ± 1.08	24.29 ± 1.77	24.17 ± 1.85	0.359	0.930	0.770
Education (years)	17.00 ± 0.37	15.57 ± 0.72	15.29 ± 0.52	15.33 ± 0.95	0.134	0.284	0.253
Participation in collision sport (years)	14.11 ± 1.45	12.17 ± 1.05	6.00 ± 2.09	7.17 ± 2.46	0.002	0.835	0.407
Athletes with a HoMC (*n*)	5	4	2	0	0.237		
Previous concussions	2.22 ± 0.55	1.71 ± 0.64	1.29 ± 0.47	0.17 ± 0.17	0.027	0.135	0.567
Symptom number	1.25 ± 0.73	4.43 ± 1.69	5.00 ± 1.54	0.33 ± 0.21	0.920	0.805	0.016
Symptom severity	2.25 ± 1.61	6.14 ± 2.50	6.86 ± 2.28	0.33 ± 0.21			

Mean (± SEM) shown. Participant demographics were assessed using a 2-way ANOVA for age, education, participation in collision sports, and previous concussions. Statistical testing of symptom number and symptom severity were performed with a 2-way MANOVA while statistical testing of a HoMC was performed with a *χ*^2^ test

*HoMC* history of multiple concussions, *SRC* sports related concussion

**Table 2:** Mean (± SEM) relCBF values for each region

	Male control	Male SRC	Female control	Female SRC
R. Gray matter	67.39 ± 3.01	62.57 ± 2.97	72.35 ± 4.75	66.60 ± 6.04
L. Gray matter	69.64 ± 3.52	64.45 ± 3.09	72.41 ± 5.08	74.85 ± 7.76
R. White matter	29.12 ± 1.32	25.99 ± 1.56	30.80 ± 2.45	29.28 ± 3.21
L. White matter	30.89 ± 1.66	26.63 ± 1.61	31.17 ± 2.39	32.24 ± 4.17
Basal ganglia	45.16 ± 1.68	40.33 ± 2.37	48.51 ± 2.89	45.68 ± 4.29

relCBF = relative cerebral blood flow, SRC = sports related concussion

### There were Significant Differences in Whole-Brain White Matter QSM Values

**Fig. 2:**
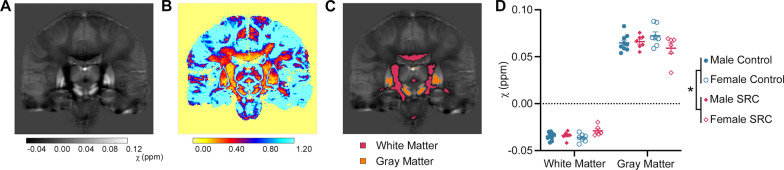
Analysis of global white and gray matter susceptibility values. **A** Mean control QSM image in study template space. Colorbar indicates susceptibility values in ppm. **B** Map of coefficient of variability. **C** Masks of global white (red, *χ* < − 0.03 ppm) and gray (orange, *χ* > 0.05 ppm) matter were generated by thresholding reliable QSM values (i.e. those voxels with coefficient of variability < 0.8) from the mean control image. **D** Two-way MANOVA revealed a significant effect of injury (*p* = 0.044, *) with post-hoc comparisons showing that SRC athletes had significantly increased susceptibility in the global white matter (Bonferroni corrected *p* = 0.020). QSM = quantitative susceptibility mapping, SRC = sports related concussion

Figure 2 presents the mean control subject QSM image in study template space (A) and the corresponding coefficient of variability image (B). The coefficient of variability image provides an estimate of reliable susceptibility within each voxel, or MRI data point. Voxels with a value below 0.8 (depicted as dark blue in Fig. 2B) were selected for further analysis and categorized as either white matter (*χ* < − 0.03 ppm) or gray matter (*χ* > 0.05 ppm) as shown in Fig. 2C [16]. Two-way MANOVA demonstrated a significant main effect of injury (*F*_2,24_ = 3.567, *p* = 0.044, Wilks’ Λ = 0.771, Fig. 2D). Pairwise comparisons with Bonferroni correction revealed that athletes with SRC had significantly increased susceptibility in the global white matter (*p* = 0.020).

### ***There was a Trend Towards Altered Straight Sinus SvO***_***2***_*** Values Following SRC***

**Fig. 3:**
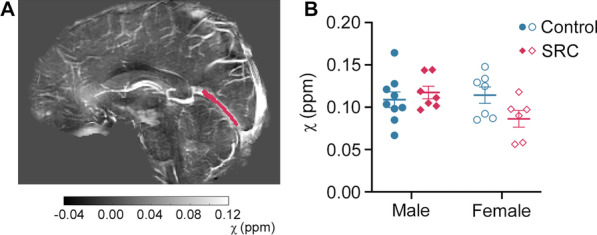
Straight sinus susceptibility as a measure of SvO_2_. **A** Representative maximum intensity projection (MIP) image for one participant with the straight sinus manually delineated in magenta. **B** Mean susceptibility values for the straight sinus for all participants. Statistical testing with 2-way ANOVA revealed a trend towards a main effect of injury (*p* = 0.061). SvO_2_ = Cerebral venous oxygen saturation

To assess straight sinus QSM values, we generated sagittal MIP images for each subject (Fig. 3A). Two-way ANOVA showed a trend towards a significant sex × injury interaction in mean susceptibility values (*F*_1,25_ = 3.858, *p* = 0.061, Fig. 3B). This trend was driven by female SRC athletes who tended to demonstrate decreased straight sinus susceptibility following SRC (*p* = 0.053). Mean (± SEM) values were: male control = 0.109 ± 0.009 ppm; male SRC = 0.117 ± 0.007 ppm; female control = 0.114 ± 0.010 ppm; and female SRC = 0.086 ± 0.010 ppm.

### Neither QSM Values, nor relCBF Values, Correlated with Symptom Number or Severity Scores

We investigated for potential relationships between symptom number and symptom severity scores and both QSM values and relCBF values. There were no significant correlations within the SRC athletes only, nor were there any significant correlations when all study participants were included following Benjamini–Hochberg false discovery rate correction (Table 3).

**Table 3:** Reported *r* and *p* values for each of the correlations between symptom number and severity and the measured relCBF and QSM values in each region

	RelCBF					*χ*		
	Right gray matter	Left gray matter	Right white matter	Left white matter	Basal ganglia	Global white matter	Global gray matter	Straight sinus *χ*
All participants (*n* = 28^a^)								
Symptom number	− 0.029, 0.885	− 0.051, 0.797	− 0.033, 0.867	− 0.006, 0.975	0.033, 0.869	− 0.377, 0.048	0.222, 0.256	0.117, 0.554
Symptom severity	0.054, 0.787	0.012, 0.951	− 0.024, 0.902	− 0.009, 0.965	0.094, 0.633	− 0.271, 0.163	0.127, 0.518	− 0.001, 0.994
SRC athletes only (*n* = 13)								
Symptom number	− 0.305, 0.311	− 0.331, 0.269	− 0.288, 0.341	− 0.276, 0.361	− 0.353, 0.237	− 0.570, 0.042	0.113, 0.713	0.375, 0.207
Symptom severity	− 0.371, 0.212	− 0.357, 0.232	− 0.409, 0.165	− 0.369, 0.215	− 0.366, 0.219	− 0.456, 0.118	− 0.003, 0.992	0.257, 0.397

No results survived Benjamini–Hochberg false discovery rate correction

^a^One subject did not report symptom number or severity

### Straight Sinus QSM Values Correlate Negatively with relCBF Values

**Fig. 4:**
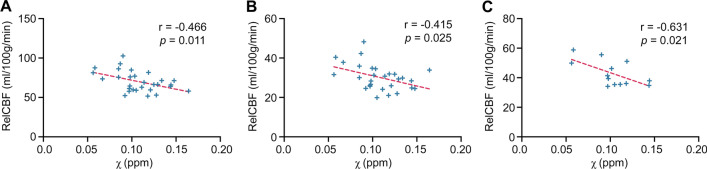
Straight sinus susceptibility values correlated with relCBF in the left hemisphere. Across all participants relCBF in the left **A** grey and **B** white matter correlated negatively with susceptibility values in the straight sinus—a measure of SvO_2_. **C** Although not reaching significance, an analysis of the SRC athletes also revealed an inverse relationship between straight sinus susceptibility and relCBF in the basal ganglia. relCBF = relative cerebral blood flow, SRC = sports related concussion, SvO_2_ = Cerebral venous oxygen saturation

Analysis of straight sinus SvO_2_ measures and relCBF in study participants revealed a significant correlation in the left grey matter (*r*_27_ = − 0.466, *p* = 0.011, Fig. 4A) and left white matter (*r*_27_ = − 0.415, *p* = 0.025, Fig. 4B). These regions also showed trends in the right hemisphere (*p* = 0.052 and *p* = 0.085, respectively).

A second analysis including just the SRC athletes revealed a relationship between straight sinus susceptibility (SvO_2_) and relCBF in the basal ganglia ROI (*r*_11_ = − 0.631, *p* = 0.021, Fig. 4C), however this did not satisfy Benjamini–Hochberg false discovery rate correction (*p* < 0.02).

## Discussion

There is widespread concern regarding the impact of repeated SRCs. Evidence from both animal [24] and human [5, 11, 18, 19] neuroimaging studies suggests that clinical recovery, i.e. the absence of symptoms, may precede physiological recovery. Here, male and female Australian rules footballers diagnosed with a recent SRC underwent MRI after being cleared to return to play following a mandatory 13-day recovery period. Australian rules football is a collision sport with tackling a key component of the game and played without compulsory protective equipment. Similar rules apply to men and women leagues, and it has one of the highest rates of SRC in Australia (80 per 100,000 participants) [25, 26].

We assessed QSM and relCBF measures in athletes with recent SRC, comparing their results to a cohort of control AFL athletes with no SRC in at least the past three months. Our findings build on previous studies at acute time points [11, 12], demonstrating global susceptibility differences at 13 days post-injury—when athletes were cleared to return to play. We showed trends for persisting hypoperfusion and increased SvO_2_ in recent SRC athletes and furthermore, significant negative correlations between SvO_2_ and relCBF measures.

A known limitation of QSM is that susceptibility values are dependent on the orientation of axons in the applied magnetic field and less reliable outside the deep grey and white matter structures [11]. As such, we assessed the variation in susceptibility between control subjects and only included those voxels that met previously defined criteria for reliability. Consistent with earlier work, we found that susceptibility was significantly increased in the global white matter and unchanged in the global grey matter following SRC [11]. This may reflect the increased vulnerability of white matter fibre bundles to the rotational forces seen in SRC although the underlying pathology driving increases in susceptibility remains to be elucidated [5, 6, 11, 12]. One interpretation is that increased white matter susceptibility reflects transient cytotoxic edema [5, 11, 12].

Hypoperfusion following mTBI [27] and SRC [16, 21, 28, 29] has been reported previously, although hyperperfusion has also been observed [14, 17, 30]. We measured relCBF values in five brain regions and found no significant differences, and only a non-significant trend, for hypoperfusion in SRC athletes compared to non-concussed controls. Meier et al., 2015 reported decreased relCBF in male athletes 1 day and 1 week after SRC compared to non-concussed controls [16]. Our results here suggest that hypoperfusion persists beyond the acute time point, consistent with a very recent study demonstrating subacute decreases in CBF that were also evident 1 year after return-to-play [18]. As hypoperfusion is thought to contribute to increased cerebral vulnerability after SRC, and potentially exacerbated outcomes following a successive concussion, these findings have important implications in return to play decision making [31].

In addition to altered cerebral perfusion, concussion also initiates cascades in cerebral metabolism [1, 2, 29]. Here, we assessed metabolic function (SvO_2_) using QSM. Taking advantage of the neuron’s almost exclusive dependence on aerobic metabolism we measured the change in susceptibility due to the relative concentrations of diamagnetic hemoglobin and paramagnetic deoxyhemoglobin [13, 14]. We found a trend of decreased susceptibility and hence increased SvO_2_ in the straight sinus of female athletes after SRC but no change in male athletes.

Previous studies have also demonstrated decreased vessel susceptibility acutely following mTBI [13, 14]. Here, we found that susceptibility in the straight sinus correlated negatively with relCBF measured in the left grey matter and white matter ROIs. Further, when this analysis was limited to the recent SRC athletes, there was a significant negative correlation between straight sinus susceptibility and relCBF in the basal ganglia ROI. Decreased susceptibility of the straight sinus implies greater levels of diamagnetic hemoglobin of the blood (i.e. elevated SvO_2_) and, in the presence of decreased relCBF, may suggest decreased oxygen consumption in the tissue [14]. Consistent with this, Champagne et al. [29] reported post-SRC group reductions in CBF and resting cerebral metabolic rate of oxygen. As oxygen extraction fraction was unchanged these results suggested acutely impaired metabolic function after SRC.

A strength of this study is the well-matched samples of male and female athletes, imaged at the same time (i.e., 13 days) post-injury. Unlike other collision sports, male and female athletes play under similar rules and without compulsory head protection, and hence they represent a unique cohort for the study of SRC. However, the small cohort size, the reliance on self-reported HoC and symptoms, and the lack of control groups without a HoC or collision sports participation are limitations of the study. Our control group (i.e., Australian footballers with a similar self-reported HoC but no recently diagnosed SRC in the past three months) is arguably the most clinically relevant in terms of assessing whether the proposed MRI biomarkers are sensitive to sub-acute SRC changes in this population; however future larger scale studies would benefit from also including control groups without a HoC and without a history of collision sport participation to provide insights into the impact of these variables. Susceptibility quantification can be confounded by partial volume effects on vessels from surrounding brain tissue, flow acceleration effects and the choice of QSM processing pipeline [32]. To best control for partial volume effects, we measured susceptibility over global gray and white matter segmentations based on a stability mask of reliable control values. We also measured SvO_2_ in the straight sinus, a large vessel approximately 5 mm in diameter [33], using maximum intensity projection images (MIPs). Furthermore, QSM images were reconstructed using nonlinear dipole inversion with Tikhonov regularization which has been shown to provide more accurate quantification of venous susceptibility than other methods [32].

## Conclusions

To mitigate the potential risk of repetitive SRC, athletes are typically withheld from competition and undertake a graded loading program, gradually increasing intensity with progression guided by the continued absence of self-reported symptoms [3]. Mandatory recovery periods have also been introduced/suggested for many sports, although there is no consensus on timing [3]. For example, in 2021, the professional Australian Football League (i.e., the AFL) updated their concussion guidelines, from a 6-day minimum recovery period to 12 days. The current study indicates that SRC can result in abnormalities in brain tissue susceptibility, relCBF and cerebral SvO_2_ that persist for at least 13 days and are present in players that have been cleared to return to play. Although these results are at a group level, and individual variability should be accounted for in the management of SRC, they do raise further concerns pertaining to the length of mandatory recovery in Australian footballers.

## Data Availability

The datasets generated and/or analysed during the current study are not publicly available due to ethical constraints but are available from the corresponding author on reasonable request.

## Abbreviations

ASL: Arterial spin labeling
CBF: Cerebral blood flow
HoC: History of concussion
HoMC: History of multiple concussions
MIP: Maximum intensity projection
MRI: Magnetic resonance imaging
mTBI: Mild traumatic brain injury
QSM: Quantitative susceptibility mapping
relCBF: Relative CBF
ROI: Region of interest
SCAT: Sports Concussion Assessment Tool
SRC: Sports related concussion
SvO_2_: Cerebral venous oxygen saturation
